# Refining the annotation of *Rhodnius prolixus* aspartic proteases A1 family genes through proteogenomics

**DOI:** 10.1016/j.crpvbd.2025.100253

**Published:** 2025-03-12

**Authors:** Radouane Ouali, Sabrina Bousbata

**Affiliations:** Laboratory of Vector-Pathogen Biology, Proteomic Platform, Department of Molecular Biology, Université Libre de Bruxelles, Gosselies, 6041, Belgium

**Keywords:** Hydrolases, Hematophagy, Proteogenomics, Chagas disease, Insect vector, *Rhodnius prolixus*

## Abstract

*Rhodnius prolixus* Stål (Hemiptera: Reduviidae: Triatominae) a hematophagous model organism and vector of Chagas disease, relies on a complex repertoire of digestive enzymes to process its blood meals. Among these, aspartic proteases from the A1 peptidase family play a crucial role in nutrient breakdown. This study aims to refine the gene annotation of the A1 peptidase family in this organism through proteogenomics. A comprehensive analysis of aspartic protease gene sequences and protein isoforms, identified by proteomics, revealed discrepancies in existing gene annotations, including the identification of novel open reading frames and the consolidation of previously separated gene sequences. Our efforts led to the correction of seven gene annotations, reducing the total count of A1 peptidase genes from 19 to 15. Notably, 11 of these genes were confirmed at the protein level, while two were supported by transcriptomic data. Furthermore, our findings highlight instances of alternative splicing, as seen in RPRC015076, where proteoforms T1IFK7 and R4G5J6 are expressed through intron retention. This study not only provides a more accurate and comprehensive genomic framework for the A1 peptidase family but also offers new insights into the functional complexity and regulation of digestive enzymes in *R. prolixus*. These findings pave the way for future studies on insect digestive biology and their potential applications in vector control strategies.

## Highlights

• Proteogenomics data were used to refine the annotation of *Rhodnius prolixus* aspartic proteases.
• Validated gene models revealed unannotated exons and corrected assembly errors.
• The number of annotated genes was reduced from 19 to 15.
• 11 of these genes were confirmed at the protein level and two were supported by transcriptomic data.

## Graphical abstract

**Figure:**
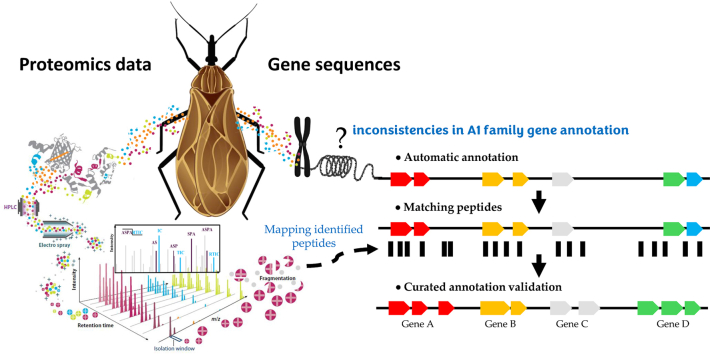

## 1 Introduction

Triatomines, commonly known as kissing bugs, are hematophagous insects belonging to the family Reduviidae, specifically the subfamily Triatominae, which includes 157 species distributed across 18 genera (Alevi et al., 2021). These insects are temporary ectoparasites that feed on a wide range of warm-blooded vertebrates, to whom they can transmit the protozoan *Trypanosoma cruzi*, the causative agent of Chagas disease, one of the most important parasitic diseases in Latin America (WHO, 2023). Triatomines are among the largest hematophagous insects, ingesting substantial amounts of blood in a single meal, often exceeding twelve times their body weight (Schaub, 2021). The blood meal, composed mainly of proteins, provides essential nutrients needed to support various metabolic processes, as well as molting, locomotion and oogenesis (Charlab and Garcia, 1997). Additionally, it supplies lipids, which are crucial for the formation of cellular structures and serve as the primary form of stored energy (Majerowicz et al., 2017), and heme, which is essential for embryogenesis and the biosynthesis of hemoproteins (Nunes-da-Fonseca et al., 2017). Blood-feeding and digestion are also intricately linked to triatomine vector competence for *T. cruzi*, as this parasite develops exclusively within the digestive tract of the vector and is transmitted *via* digestive byproducts (Garcia et al., 2010; Schaub, 2021).

Proteins are released following the lysis of erythrocytes in the anterior midgut (AM) (Azambuja et al., 1983). The digestion of blood proteins is primarily mediated by lysosomal cathepsin-like enzymes (Houseman and Downe, 1982; Terra et al., 2019; Henriques et al., 2021; Ouali and Bousbata, 2024), particularly aspartic proteases from the A1 family. Notably, previous studies have shown that ingesting pepstatin A, a specific inhibitor of aspartic proteases, significantly impedes blood digestion in the midgut of *Rhodnius prolixus*. This inhibition correlates with a marked delay in molting across all nymphal instars and has a profound impact on oogenesis in adult stages (Garcia et al., 1981), underscoring the crucial role of these enzymes. Our recent work also demonstrated that treatment of digestive contents with pepstatin A effectively abrogated hemoglobin digestion, indicating that its initial cleavage by these enzymes is essential for subsequent digestion by other midgut-associated peptidases (Ouali and Bousbata, 2024).

Aspartic proteases are widely conserved enzymes that play essential roles in protein degradation, maturation, and virulence across diverse organisms, including vertebrates, fungi, and parasites (Davies, 1990). All the peptidases in family A1 are endopeptidases, mainly active at acidic pH, and with two aspartic residues forming the catalytic dyad, responsible for catalytic activity. They are initially produced as inactive precursors with an N-terminal signal peptide and a propeptide. They specifically target peptide bonds flanked by residues with large hydrophobic side chains (Dunn and Hung, 2000). In many parasites, including malaria pathogens, platyhelminths, nematodes, and ticks, aspartic proteases function within multienzyme hemoglobinolytic complexes, initiating hemoglobin degradation before further processing by other digestive peptidases. Their inhibition represents a promising strategy to disrupt amino acid acquisition, essential for parasite survival (Sojka et al., 2016).

The genome-wide mapping of peptidases in *R*. *prolixus* identified nineteen genes belonging to the A1 peptidase family (Henriques et al., 2017). Analysis of transcript expression in digestive tissues revealed 14 aspartic proteases, predominantly expressed in the AM, with 13 members identified in this tissue (Ribeiro et al., 2014). Among these, ten were validated at the protein level as midgut-associated peptidases (Ouali et al., 2020), and notably, the expression of seven isoforms in the AM was induced post-prandially (Ouali et al., 2021). Furthermore, 18 A1 genes were identified to be expressed in the AM using qRT-PCR, and a temporal analysis of their expression profiles revealed differential expression throughout the digestive process (Henriques et al., 2021).

A deep profiling of midgut-associated aspartic proteases, performed through pepstatin A affinity chromatography purification from the AM at various time points, followed by quantitative proteomic analysis, enabled the reconstruction of their expression patterns during digestion, revealing a total of 27 protein isoforms (Ouali and Bousbata, 2024). Strikingly, many of these isoforms displayed similar expression profiles. Furthermore, significant variations in the lengths of some isoforms strongly suggest that they may represent fragmented portions of a single protein, likely derived from a common gene. Through sequence alignment of these isoform sequences and detailed manual genomic sequence analysis, we uncovered inconsistencies in the existing gene annotations.

In this context, this study aims to refine the gene annotation in this peptidase family by leveraging MS/MS-sequenced peptides identified in previous proteomics investigations. Detected peptides from tandem mass spectrometry were systematically mapped onto the corresponding nucleotide sequences, allowing for a thorough revision of genes exhibiting anomalies, including the identification of a previously unannotated open reading frame and the consolidation of sequences originally annotated as separate genes.

## 2 Materials and methods

### 2.1 Reannotation of aspartic proteases A1 family genes by means of proteomics

The A1 family genes identified in the *R. prolixus* genome (Mesquita et al., 2015) are cross-referenced in the VectorBase database (https://vectorbase.org/vectorbase/app/). The gene and contig sequences, along with their locations, were retrieved from the database (accessed in March 2023). The previously transcript sequences identified by Ribeiro et al. (2014) can be found on GenBank. Predicted Protein sequences encoded by these genes are accessible on UniProt (https://www.uniprot.org/, accessed in March 2023).

Genomic sequences of *R*. *prolixus* aspartyl proteases were subjected to detailed manual annotation review, focusing on both intronic and exonic regions. This analysis aimed to identify annotation discrepancies, including the presence or absence of stop codons, misannotation of intronic regions as exons, and erroneous annotation of intergenic regions. Manual sequence inspection was informed by prior proteomic data. The peptides identified by mass spectrometry in our previous proteomic investigations (Ouali et al., 2020; Ouali and Bousbata, 2024) were retrieved from the ProteomeXchange Consortium (PXD019150 and PXD044628, respectively) and are detailed in Supplementary file S1.

### 2.2 Sequence similarity searches and protein sequence alignments

Sequence similarity searches were performed using the BLASTn tool, targeting available transcript and genomic sequences on VectorBase (https://vectorbase.org). The searches were carried out with default parameters. For protein sequence alignments, the sequences were retrieved from UniProt (https://www.uniprot.org) and aligned using the default settings to assess sequence conservation and identify homologous proteins.

## 3 Results and discussion

**Fig. 1:**
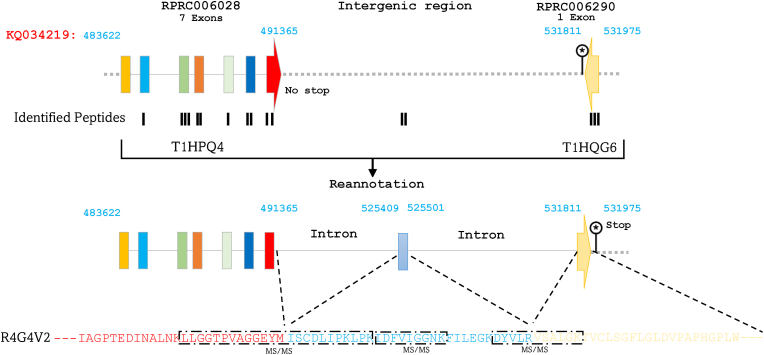
Alignment of T1HPQ4 and T1HQG6 revealed the full-length protein R4G4V2 and identified a novel exon. The isoforms T1HPQ4 and T1HQG6, encoded by RPRC006028 and RPRC006290, were aligned to reconstruct the full-length protein R4G4V2. Genomic analysis of KQ034219 uncovered a previously unannotated exon (525,409–525,501 bp) and redefined RPRC006290 as part of RPRC006028 on the positive strand. Peptide mapping validates this revised annotation, covering the seventh exon of RPRC006028, the novel exon, and an additional intermediate region. Exons are represented by colored rectangles, with the terminal exon indicated by an arrow. Introns are depicted by continuous lines, while intergenic regions are shown as dashed lines. MS/MS-identified peptides are represented by black rectangles aligned with their corresponding genomic sequences.

The gene RPRC006028, comprising 7 exons and lacking a stop codon, encodes for T1HPQ4, and RPRC006290, consisting of a single exon, coding T1HQG6 are localized in the genomic region KQ034219. Strikingly, aligning the two isoforms encoded by these two genes, revealed that each of them represents a fragment of the full-length protein R4G4V2 (Fig. 1). A more detailed analysis of R4G4V2, in comparison to the genomic sequence KQ034219 (483,622–531,975 bp), indicates the presence of an unannotated exon within the presumed intergenic sequence, precisely located between 525,409 and 525,501 bp and encodes a part of the protein. Additionally, it has been discovered that the gene RPRC006290, originally annotated on the negative strand, is in fact an exon of the gene RPRC006028 located on the positive strand. This revised annotation is substantiated by the sequenced peptides that encompass a portion encoded by the seventh exon of the gene alias RPRC006028, along with the newly reannotated exon within the intergenic sequence, and another between the latter and RPRC0062906. Consequently, it can be inferred that the region KQ034219 (483,622–531,975 bp) contains a single gene consisting of 9 exons, which encode for R4G4V2 (Fig. 1 and Supplementary file S2). R4G4V2 expression in the midgut was validated at both the transcriptional (Ribeiro et al., 2014; Henriques et al., 2021) and proteomic levels (Ouali et al., 2020; Ouali and Bousbata, 2024), confirming the presence of corresponding mRNA and protein products. Corresponding mRNA was also detected in the ovaries and oocytes (Coelho et al., 2021; de Almeida et al., 2023), indicating their involvement in yolk degradation and the reproductive processes in this vector.

**Fig. 2:**
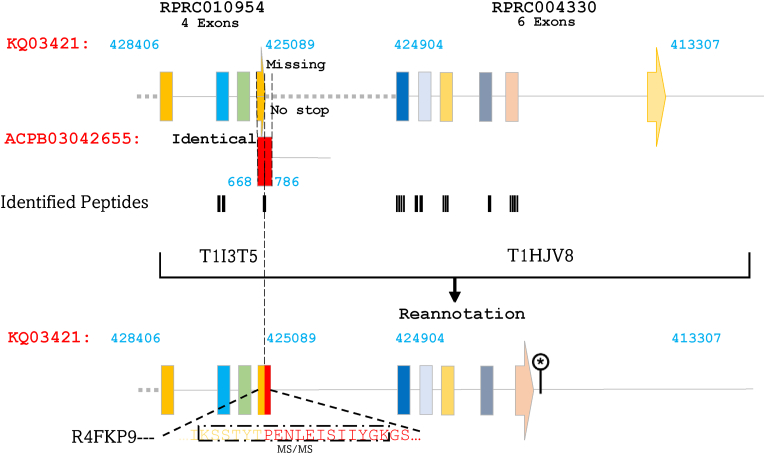
Alignment of T1I3T5 and T1HJV8 confirms the full-length protein R4FKP9 and resolves assembly errors. Isoforms T1I3T5 and T1HJV8 were aligned to reconstruct R4FKP9, encoded by a single gene within KQ03421 (413,307–428,406 bp) on the negative strand. A missing exon fragment in RPRC010954, due to assembly errors, was recovered on contig ACPB03042655 (668–786 bp). MS/MS peptide evidence validates this correction and confirms the revised gene structure. Exons are represented by colored rectangles, with the terminal exon indicated by an arrow. Introns are depicted by continuous lines, while intergenic regions are shown as dashed lines. MS/MS-identified peptides are represented by black rectangles aligned with their corresponding genomic sequences.

This pertains to the genes RPRC010954, encoding for T1I3T5 and RPRC004330 encoding for T1HJV8, located on the region KQ03421 (413,307–428,406 bp) on the negative strand. Investigation of the sequences of the isoforms encoded by these two presumed genes revealed that each is merely a fragment of the full-length protein R4FKP9 (Fig. 2 and Supplementary file S2), encoded by a single gene located between 413,307 and 428,406 bp. Furthermore, we have shed light on the fact that the last exon of the presumed gene RPRC010954 lacks a fragment, certainly due to a sequencing/assembling issues. This fragment was found on the contig ACPB03042655 (668–786 bp), which shares identity with the corresponding region of this exon on KQ03421. The identification of the peptide by MS/MS commonly encoded by both fragments, as shown in Fig. 2, confirms the annotation. R4FKP9 mRNA and protein were identified in the digestive tract of *R. prolixus* (Ribeiro et al., 2014; Ouali et al., 2020; Henriques et al., 2021). Furthermore, its expression in the AM is upregulated in response to infection with *T. cruzi* (Ouali et al., 2024).

**Fig. 3:**
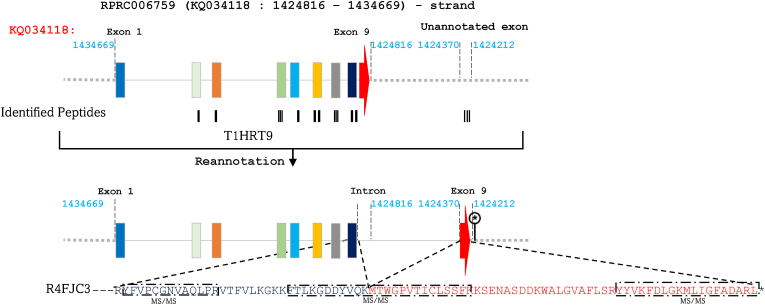
Identification of a novel exon encoding the C-terminal region of R4FJC3 and correction of RPRC006759 gene structure. T1HRT9, encoded by RPRC006759 (KQ034118: 51,424,816–1,434,669 bp) on the negative strand, corresponds to R4FJC3, which lacks a C-terminal region. BLAST analysis revealed an unannotated exon (1,424,212–1,424,370 bp) encoding this region and a stop codon. MS/MS data validate this annotation, linking the eighth exon of RPRC006759 to the novel exon, while the previously annotated ninth exon is corrected as a misclassified intron. Exons are represented by colored rectangles, with the terminal exon indicated by an arrow. Introns are depicted by continuous lines, while intergenic regions are shown as dashed lines. MS/MS-identified peptides are represented by black rectangles aligned with their corresponding genomic sequences.

In a similar vein, alignment of the different aspartic protease isoforms revealed that T1HRT9 coded by RPRC006759 located on the genomic region KQ034118 (51,424,816–1,434,669 bp) on the negative strand aligns perfectly with R4FJC3, which additionally possesses a missing C-terminal region (Fig. 3). Furthermore, RPRC006759 lacks a stop codon. BLAST analysis between the mRNA sequence encoding R4FJC3 and *R. prolixus* genomic sequence indicates that the C-terminal region of this protein is encoded by an unannotated exon, followed by stop codon at 1,424,212–1,424,370 bp in the presumed intergenic region (Fig. 3). Through MS/MS analysis, we have identified a peptide commonly encoded by the eight exon of RPRC006759 and the newly annotated exon, thereby confirming this annotation (Fig. 3). Moreover, our study revealed that what was previously annotated as the ninth exon of RPRC006759 is, in fact, a misclassified intron (Fig. 3 and Supplementary file S2).

**Fig. 4:**
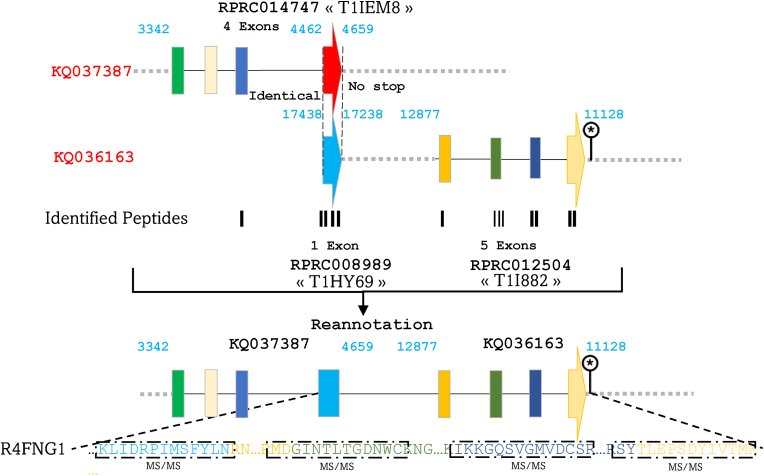
Correction of gene annotations for RPRC014747, RPRC008989, and RPRC012504 coding R4FNG1. RPRC014747 encoding T1EM8 lacks a stop codon, its final exon is identical to the sole exon of RPRC008989, which encodes T1HY69. Sequence analysis revealed that T1HY69 is a fragment of T1EM8, which in turn is part of the full-length R4FNG1 protein. The remaining portion corresponds to T1I882, encoded by RPRC012504 downstream of RPRC008989. These three genes, initially considered independent, are redefined as components of a single gene encoding R4FNG1. Exons are represented by colored rectangles, with the terminal exon indicated by an arrow. Introns are depicted by continuous lines, while intergenic regions are shown as dashed lines. MS/MS-identified peptides are represented by black rectangles aligned with their corresponding genomic sequences.

**Fig. 5:**
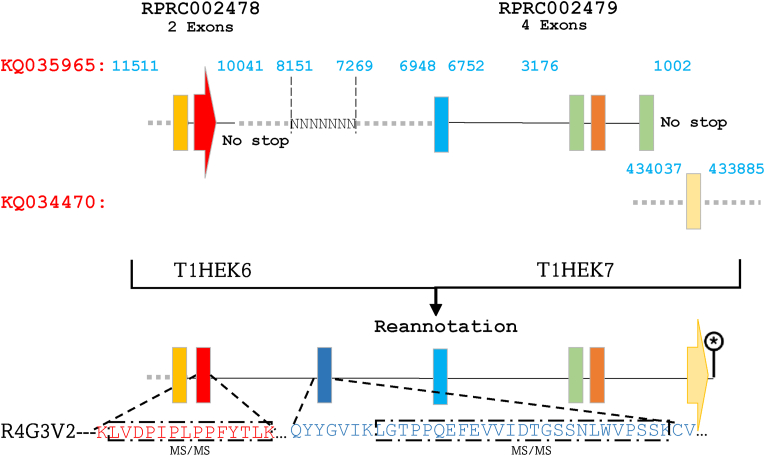
Revision of the gene annotation encoding the protein R4G3V2. T1HEK6 and T1HEK7, encoded by RPRC002478 and RPRC002479, are truncated fragments of R4G3V2. A missing protein fragment is encoded by an unannotated exon in the intergenic region, confirmed by MS-identified peptides. A missing C-terminal exon, located on contig KQ034470 (433,885–434,037 bp), indicates a genome assembly issue. Exons are represented by colored rectangles, with the terminal exon indicated by an arrow. Introns are depicted by continuous lines, while intergenic regions are shown as dashed lines.

On the other hand, we noticed that RPRC014747, located on KQ037387 (3342–4659 bp) and comprising four exons, codes for T1EM8 (22 kDa) does not contain a stop codon (Fig. 4). Furthermore, its last exon shows absolute identity with the sole exon of the gene alias RPRC008989, located between 17,238 and 17,438 bp of region KQ036163 and encoding for T1HY69. Sequence alignment discloses that T1HY69 merely constitutes a fragment of T1EM8, which is a fragment of the full-length R4FNG1 protein (Fig. 4 and Supplementary file S2). The other part (22.4 kDa) corresponds to T1I882, encoded by the presumed gene RPRC012504 located downstream of the gene RPRC008989 at 11,128–12,877 bp. Consequently, RPRC014747, RPRC008989, and RPRC012504, initially annotated as independent genes, are indeed integral entities of the same gene encoding for the R4FNG1 protein. The sequencing of peptides commonly encoded by these entities constitutes formal evidence for this annotation (Supplementary file S2). This protein was identified in the AM, and its post-prandial upregulated expression suggests a role in the initiation of the digestive process (Ouali et al., 2021). In addition, we demonstrated an induction of its expression during the first hours post-infection with *T. cruzi* (Ouali et al., 2024). Likewise, it is apparent that T1HEK6 (8.5 kDa) and T1HEK7 (21.5 kDa), encoded respectively by RPRC002478 and RPRC002479, which are adjacently positioned on the negative strand within region KQ035965 (949–11,511 bp), represent truncated fragments of the protein R4G3V2 (Fig. 5). Indeed, observation of these gene sequences revealed a conspicuous absence of stop codon at the end of these genes. Furthermore, this scrutiny unveils that a portion of the R4G3V2 protein is encoded by an intriguingly unannotated exon nestled within the intergenic region at 7269–8151 bp (Fig. 5 and Supplementary file S2).

The genomic sequence corresponding to this exon is inaccurately sequenced, but could be predicted through the sequence of R4G3V2 as well as the mRNA sequence of Rp-10596 (Ribeiro et al., 2014) encoded by the complete 7-exons containing gene. The MS sequencing of a supporting peptide encoded by the newly annotated exon firmly validates the amalgamation of the hypothetical gene entities, substantiating this all-encompassing annotation as a single gene (Fig. 5). Moreover, we noticed that the exon encoding the C-terminal portion of R4G3V2 is lacking (Supplementary file S2). A BLAST search of the mRNA sequence for this protein revealed that this missing exon was located on the contig KQ034470 (433,885–434,037 bp). This fragmentation likely reflects an issue in the genome assembly.

**Fig. 6:**
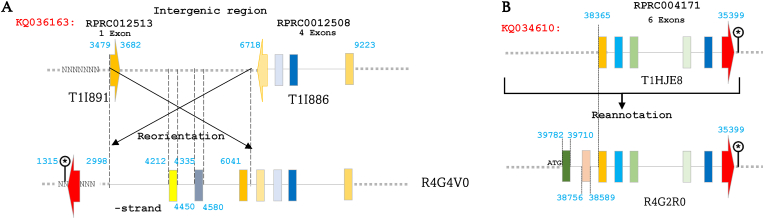
Reannotation of genomic regions KQ036163 and KQ034610 clarifies gene structures for R4G4V0 and R4G2R0 proteins. **A** The inversion of KQ036163 (3479–6718 bp) redefined RPRC012513 (T1I891) as a fragment of R4G4V0. Reannotation on the negative strand identified three new exons (1315–4580 bp), confirmed by the mRNA sequence. **B** RPRC004171, encoding T1HJE8 was found identical to R4G2R0. BLAST analysis of the mRNA sequence revealed two upstream exons, reclassifying RPRC004171 as a gene with eight exons encoding R4G2R0. Exons are represented by colored rectangles, with the terminal exon indicated by an arrow. Introns are depicted by continuous lines, while intergenic regions are shown as dashed lines.

Among annotation errors detected during gene and protein sequences investigation, the inversion of the fragment 3479–6718 bp of the genomic region KQ036163 has been detected. Initially, this region was annotated with the gene alias RPRC012513 (coding for T1I891) on the positive strand. However, an in-depth investigation of T1I891 sequence revealed that it is a fragment of the protein R4G4V0 (Fig. 6A and Supplementary file S2). Moreover, the C-terminal part of R4G4V0 shows total identity to protein T1I886, encoded by RPRC0012508 located downstream of RPRC012513 (6718–9223 bp). Upon reannotating the inverted region on the negative strand, three new exons were designated between 1315 and 4580 bp (Fig. 6A). Despite R4G4V0 has not been identified by proteomics, the correct orientation was validated through the identification of the mRNA RP-8066 coded by a gene grouping presuppositional RPRC0012508 and RPRC012513 genes (Ribeiro et al., 2014). Additionally, the genomic region between 1315 and 2998 bp was not accurately sequenced, but we were able to ascertain it through the mRNA RP-8066 sequence. In the same context, RPRC004171, positioned at KQ034610 (35,399–38,365 bp), revealed annotation issues. In fact, the hypothetical protein T1HJE8 (36 kDa) encoded by this gene exhibits an absolute identity with protein R4G2R0 (45.3 kDa). Blasting the mRNA sequence encoded by this gene (RP-6421) against *R. prolixus* genome revealed the existence of two exons upstream of the presumed RPRC004171 (Fig. 6B). Consequently, we conclude that this gene comprises 8 exons encoding the complete R4G2R0 protein. The protein named T1HJE8 does not exist as an independent isoform but rather represents only a fragment of the complete protein.

**Fig. 7:**
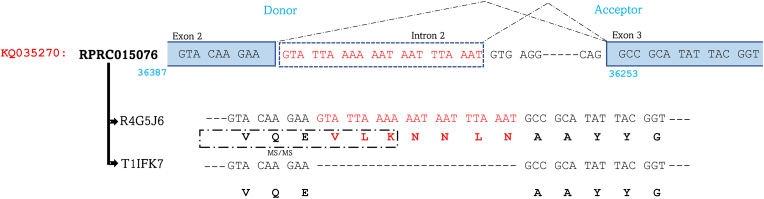
Intron retention mechanism in RPRC015076 generates R4G5J6 and T1IFK7 proteoforms. RPRC015076, located in KQ035270, encodes two isoforms: R4G5J6 and T1IFK7. Sequence analysis shows T1IFK7 is the conventional isoform, while R4G5J6 arises through intron retention, incorporating 7 additional amino acids. The identification of donor and acceptor sites, along with a shared peptide encoded by the end of exon 2 and retained intron 2, confirms this mechanism of isoform diversification.

Sequence alignment revealed that RPRC015076, which is located on the genomic sequence KQ035270 encodes two distinct proteoforms simultaneously: R4G5J6 and T1IFK7. Through meticulous sequence analysis of both forms, we have discerned that T1IFK7 is conventionally encoded by this gene. However, the intriguing expression of R4G5J6 involves a captivating process of intron retention. Remarkably, donor and acceptor sites were identified as indicated in Fig. 7. This facilitates the coding of this isoform with 7 additional amino acids, resulting in subtle changes in its sequence. Crucially, the identification of a shared peptide, encoded jointly by the end of exon 2 and the retained part of intron 2, unequivocally confirms the implementation of this intricate mechanism governing isoform diversification (Fig. 7). Both proteoforms are expressed in the AM and are upregulated in response to blood ingestion (Ouali et al., 2021; Ouali and Bousbata, 2024)

Attributing accurate annotations to genes RPRC01175 and RPRC012487, which encode the hypothetical proteins T1I633 (15.8 kDa) and T1I865 (7.5 kDa), respectively, has proven to be a challenging endeavor. Indeed, the expression of these genes has never been detected at the protein level, and the absence of unique sequenced peptides corresponding to their respective forms precludes their categorization as functional entities. Nevertheless, it is plausible that these fragments might represent segments of a singular gene. The same holds true for RPRC012664 encoding T1I8P2 (35.5 kDa) and RPRC002696 encoding T1HF74 (11.7 kDa). No discernible evidence at the protein level has enabled us to ascertain the functional status of these two putative genes or to rectify their current annotation.

Leveraging the expression profiles of distinct isoforms, along with the complete set of sequenced peptides, facilitated the rectification of gene annotations within the A1 family. This corrective endeavor led to the accurate annotation of seven genes. Consequently, this correction resulted in the inference that the insect genome encompasses 15 A1 genes (Table 1) and not 19, as previously indicated (Henriques et al., 2017). Eleven have been substantiated at the protein level, and two genes were demonstrated solely through transcriptomic evidence. Regarding the putative genes RPRC012664 and RPRC002696, no evidence of their expression exists. In addition, RPRC011752 and RPRC012487 cannot be confidently regarded as individual genes. Indeed, while lacking evidence of their expression, it is highly improbable that they serve as functional entities; rather, they seemingly represent fragments of other genes, potentially disrupted during the sequencing process. Furthermore, we have illuminated the occurrence of alternative splicing at donor or acceptor sites positioned within a narrow span of nucleotides. This event engenders subtle modifications in T1IFK7 and R4G5J6 isoforms concurrently expressed by RPRC015076.

**Table 1:** *Rhodnius prolixus* aspartic proteases genes and proteins.

A1 family genes	Genomic location	Digestive transcripts ID	Proteins Uniprot ID	Mass (kDa)	Figure
RPRC015079	KQ035270:22,283 … 29,217	Rp-6850	R4G5J4	43,622	
RPRC015076	KQ035270:31,145 … 38,451	Rp-6846	T1IFK7/R4G5J6	43,383/44,846	Fig. 7
RPRC006028-RPRC006290	KQ034219:483,622 … 531,975	Rp-1760	R4G4V2	42,388	Fig. 1
RPRC006759	KQ034118:1,424,212 … 1,434,669	RP-3415	R4FJC3	45,508	Fig. 3
RPRC004330-RPRC010954	KQ03421:413,307 … 428,406	RP-7417	R4FKP9	43,820	Fig. 2
RPRC002478-RPRC002479	KQ035965:948 … 11,511	Rp-10596	R4G3V2	44,082	Fig. 5
RPRC014747-RPRC012504-RPRC008989	KQ037387:3342 … 4462-KQ036163:11,128 … 17,438	Rp-2814	R4FNG1	43,137	Fig. 4
RPRC012508-RPRC012513	KQ036163:1315 … 9223	RP-8066	R4G4V0	43,233	Fig. 6A
RPRC004171	KQ034610:35,399 … 39,782	RP-6421	R4G2R0	45,341	Fig. 6B
RPRC012785	KQ034079:4811 … 10,271	Rp-2217	R4FNN7	44,222	
RPRC015082	KQ035270:12,987 … 20,613	RP-82226	R4FP52	43,408	
RPRC006698	KQ034534:71,381 … 77,412	RP-5007	R4FMP1	43,907	
RPRC012786	KQ034079:13,511 … 18,584		T1I914	42,923	
RPRC012664	KQ035425:10,005 … 15,720		T1I8P2 a	35,511	
RPRC002696	KQ034126:569,709 … 570,120		T1HF74 b	11,732	
RPRC011752	ACPB03043556:28 … 700		T1I633 b	15,897	
RPRC012487	ACPB03042715:162 … 362		T1I865 b	7486	

a Missing N-terminal.

b Truncated.

## 4 Conclusions

The diversity and multiplicity of digestive protease genes in *R. prolixus* is a common feature among hematophagous insects, including mosquitoes (Isoe et al., 2009) and sand flies (Abrudan et al., 2013), where multiple protease isoforms are expressed to ensure efficient digestion of blood meals. This redundancy and functional versatility allow these insects to adapt to varying physiological conditions and optimize proteolytic activity. Alternative splicing further enhances this adaptability by generating enzyme proteoforms with distinct substrate specificities, fine-tuning their digestive processes. Correcting these gene inaccuracies not only improves gene annotations but also provides valuable insights into the molecular mechanisms of digestion and host adaptation in blood-feeding insects. By understanding the full diversity of proteases, we can better identify potential targets for vector control strategies. These newly identified protease variants could serve as critical points of intervention, offering novel avenues for disrupting the triatomine’s ability to digest blood or interact with pathogens, thereby reducing transmission efficiency and potentially controlling the spread of diseases.

## CRediT authorship contribution statement

**Radouane Ouali:** Conceptualization, Methodology, Visualization, Supervision, Funding acquisition, Writing – original draft, Writing – review & editing. **Sabrina Bousbata:** Supervision, Funding acquisition, Writing – review & editing.

## Ethical approval

Not applicable.

## Funding

This research was funded by the Fonds de la Recherche Scientifique of Belgium (FNRS), grant number J.0019.20/22, awarded to Sabrina Bousbata. Radouane Ouali is an FNRS associate researcher. Grants from the Association des Amis des Instituts Pasteur à Bruxelles, and from the De Meurs-François Foundation have been attributed to Radouane Ouali.

## Declaration of competing interests

The authors declare that they have no known competing financial interests or personal relationships that could have appeared to influence the work reported in this paper.

## Data Availability

All data generated or analyzed during this study are included in this published article and its supplementary files.

## Appendix A Supplementary data

Multimedia component 1 Supplementary file S1. List of peptides derived from aspartic proteases identified through tandem mass spectrometry.

Multimedia component 2 Supplementary file S2. mRNA and protein sequences of revised genes.
