# Supplementary material for: Refining the annotation of Rhodnius prolixus aspartic proteases A1 family genes through proteogenomics
Source: Curr Res Parasitol Vector Borne Dis. 2025 Mar 12;7:100253. doi: 10.1016/j.crpvbd.2025.100253 (PMC11978366; doi:10.1016/j.crpvbd.2025.100253)
Supplement: Multimedia component 2 — Supplementary file S2. mRNA and protein sequences of revised genes. [file mmc2.pdf]

1) R4G4V2

- **mRNA Sequence (Introns spliced out)**

RPRC006028

ATGTTAGCACATACGCTGATATTAATATCATCGCTCTGTGGTGTTCTCACAGGAAGCGATAATCTCGTAAGAGTA
CCATTAACAAAAATACAATCAGCAAGGAGAACATTTCAAGAAGTTGGAACTTCAGTTAAACAACTTTCAATTAAG
TATGCCGGCAACAGTGTTGGTATAGATGGTCCATTCCCAGAACCCCTATCAAATTACCTTGATGCCCAGTATTAT
GGTCCAATTACTCTGGGATCACCGCCACAGTCTTTCAGAGTCGTTTTTGATACAGGTTCTTCCAATTTATGGGTG
CCTTCGAAGAAATGCTCAAAATTCAATATTGCTTGCTGGGTTCATCGCAAATATGATAGTTCTAGTTCTAAAACA
TATGTGCCTAATGGAGAAAAATTTGCCATTCAATATGGAAGTGGAAGTCTTTCTGGTTTTCTGTCGCAGGATCAT
TTGACTATTGGAGGCATCACTGTTGCTAATCAAACATTTGCAGAAGCTGTAAATGAACCAGGAATGGTATTTGTA
GCAGCTAAATTTGATGGTATTCTTGGTCTTGGTTATGACACTATATCTGTAGATAAAGTTACACCTCCATTTTAC
AATATGTATAAGCAAGGAGTGGTTCAGACACCAGTATTTTCTTTTTATCTCAATAGGGATCCCGCAGCAAGTGTT
GGCGGTGAAATCATATTCGGAGGTTCGGATCCCGAGAAATATGTTGGTGATTTCACTTACGTACCCGTAAATAAA
CAAGGATACTGGCAATTTAGCATGGATTCAGTTATAGCAAATGGAAAGACTTTCTGCAAAGGGGGTTGCCAAGCC
ATTGCTGATACAGGTACAAGCTTGATTGCTGGCCCTACCGAGGATATTAATGCTCTCAATAAGTTACTTGGAGGT
ACTCCAGTAGCAGGAGGAGAATACATGATATCCTGTGATTTAATACCAAAATTACCTAAAATTGATTTTGTTATT
GGAGGAAACAAATTTATATTGGAAGGAAAGGATTACGTCCTTAGAGTCTCTGCATTGGCAAGACTGTTTGCTTA
TCCGGCTTTCTTGGGTTAGACGTTCCTGCGCCACATGGCCCGCTATGGATTCTTGGCGATGTCTTCATTGGACGA
TTCTATACCGAATTCGATCTAGGCAATAATAGAGTGGGATTCGCATTGTCCAAAGAATAA

Missing exon

RPRC006290

mRNA sequence (intron spliced out) of R4G4V2. Exons are color-coded for distinction, with odd-numbered exons in blue and even-numbered exons in gray. The genes encoding each fragment are indicated.

- **Protein sequence**

MS/MS identified peptides

Encoded commonly by two exons

MLAHTLILISSLCGVLTGSDNLVRVPLTKIQSAR**RTFQEVGTSVK**QLSIKYAGNSVGIDGPF
PEPLSNYLDAQYYGPITLGSPPQSFR**VVFDTGSSNLWVPSK**K**CSKFNIACWVHR**KYDSSSSK
TYVPNGEKFAIQYGSGSLSGFLSQDHLTIGGITVANQTFAEAVNEPGMVFVAAK**FDGILGLG**
YDTISVDKVTPPFYNMYKQGVVQTPVFSFYLNR**DPAASVGGEIIFGGSDPEK**YVGDFTYVPV
NK**QGYWQFSMDSVIANGK**TFCK**GGCQAIADTGTSLIAGPTEDINALNK**LLGGTPVAGGEYMI
SCDLIPKLPK**IDFVIGGNKFILEGKDYVLR**VSALGKTVCLSGFLGLDVPAPHGPLWILGDVF
IGR**FYTEFDLGNNR**VGFALSKE*

Amino acid sequence of R4G4V2. Peptides sequenced by MS/MS are highlighted in bold and underlined. Peptides encoded jointly by two exons are indicated.

- **Sequence alignment**

```
R4G4V2 MLAHTLILISSLCGVLTGSDNLVRVPLTKIQSARRTFQEVGTSVKQLSIKYAGNSVGIDG 60
T1HPQ4 MLAHTLILISSLCGVLTGSDNLVRVPLTKIQSARRTFQEVGTSVKQLSIKYAGNSVGIDG 60
T1HQG6 ------------------------------------------------------------ 0

R4G4V2 PFPEPLSNYLDAQYYGPITLGSPPQSFRVVFDTGSSNLWVPSKKCSKFNIACWVHRKYDS 120
T1HPQ4 PFPEPLSNYLDAQYYGPITLGSPPQSFRVVFDTGSSNLWVPSKKCSKFNIACWVHRKYDS 120
T1HQG6 ------------------------------------------------------------ 0

R4G4V2 SSSKTYVPNGEKFAIQYGSGSLSGFLSQDHLTIGGITVANQTFAEAVNEPGMVFVAAKFD 180
T1HPQ4 SSSKTYVPNGEKFAIQYGSGSLSGFLSQDHLTIGGITVANQTFAEAVNEPGMVFVAAKFD 180
T1HQG6 ------------------------------------------------------------ 0

R4G4V2 GILGLGYDTISVDKVTPPFYNMYKQGVVQTPVFSFYLNRDPAASVGGEIIFGGSDPEKYV 240
T1HPQ4 GILGLGYDTISVDKVTPPFYNMYKQGVVQTPVFSFYLNRDPAASVGGEIIFGGSDPEKYV 240
T1HQG6 ------------------------------------------------------------ 0

R4G4V2 GDFTYVPVNKQGYWQFSMDSVIANGKTFCKGGCQAIADTGTSLIAGPTEDINALNKLLGG 300
T1HPQ4 GDFTYVPVNKQGYWQFSMDSVIANGKTFCKGGCQAIADTGTSLIAGPTEDINALNKLLGG 300
T1HQG6 ------------------------------------------------------------ 0

R4G4V2 TPVAGGEYMISCDLIPKLPKIDFVIGGNKFILEGKDYVLRVSALGKTVCLSGFLGLDVPA 360
T1HPQ4 TPVAGGEYM--------------------------------------------------- 309
T1HQG6 ----------------------------------------VSALGKTVCLSGFLGLDVPA 20

R4G4V2 PHGPLWILGDVFIGRFYTEFDLGNNRVGFALSKE 394
T1HPQ4 ---------------------------------- 309
T1HQG6 PHGPLWILGDVFIGRFYTEFDLGNNRVGFALSKE 54
```

2) R4FKP9

- **mRNA sequence**

RPRC010954

ACPB03042655

RPRC004330

ATGAAGTTGATATTCGTGTTTCTGGTCTTCTCTCCCATCTTTTATGGATCAGCAGCACATGTTGGAAAGGTTCAA
CTTTATCGAAGACAACCAAAATCTTACGAACAATATTTGAATGAAAACATCGCCTATCGGGACGCTCTCTCCCAG
CTGATCAATCTAAAGAGTTCTGGCCATAGCAGTGATACAGTAGATCTGCGCAATTTTGCCAATTCCCAATATTAT
GGCTCCATATCTATTGGAACACCACCTCAAAGTTTTGAAATATTATTCGATACTGGGTCCACCTTGTTATGGGTG
CCTTCATTCGACTGCTTCAGCCCTGCTTGTATGACCCACCGAAAGTATGACCACATAAAATCAAGCACTTATACA
CCAGAAAATTTGGAAATTAGCATAATCTATGGCAAAGGCAGTATGACGGGAATCTTGGGTAGAGATACCTTACAG
ATTGGAAATTTCACGGTAAGGCGACAACAATTTGCTGAAGCAACCAAAGAACCTGGGAATGCCTTTTTACATTCC
CAATTTGATGGAATATTGGGCATGGCGTTTCCAACTGCAGGTTCAAAAGTTGAACCAGTCTTCCATACACTGATA
AAACAAGGACTGGTTGAAGATCGTATATTTTCTTTTTTTCTTAACAGAGATCCTAACGGAGATTTGGGAGGCGAA
ATGATCTTAGGTGGCTGGAATGATTATTACTTCGATTCGTCAAAAATTAACTACATTCGTCTTTCTAAGGTGGAT
AAATGGCAGTTTAAAGTGGACTCCATTTCAAGCACAAATAAAACAATGTTTTGCAAAGATAGTTGCGAAGCTATT
GCAGATACAGGCACCACAATGATTATTGGTCCTACTGATGAAATCGCAGCCATACACAAATATATCGGTGCTGTT
CAAAACTACGGTATGGGAACAGTTAAGTGTAAAGACGTCCCCAATCTTCCGCCCATTACATTCCTCATCAATGGC
ACGGAGTATGTCCTGAAAGGAAAAGATTACATCCTTAATTTTGATTACGATCCCTACAACTGTATAACCGGCTTC
TCTGGCGTAGAATTAAAAGGAAAGCCCTGGATTTTAGGCGATGTCTTTTTGCGCAAGTTTTACACCATCTTCAAC
GTTCAAGATAAAGCTGTAGCCTTCGCTACATTAAACAAGTTGCTTTAA

mRNA sequence of R4FKP9. Exons are color-coded for distinction, with odd-numbered exons in blue and even-numbered exons in gray. The genes encoding each fragment are indicated.

- **Protein sequence**

MS/MS identified peptides

Encoded commonly by two exons

T1I3T5

T1HJV8

MKLIFVFLVFSPIFYGSAAHVGKVQLYRRQPK**SYEQYLNENIAYR** **DALSQLINLK** SSGHSSD

TVDLRNFANSQYYGSISIGTPPQSFEILFDTGSTLLWVPSFDCFSPACMTHRKYDHIK**SSTY**

TPENLEISIIYGKGSMTGILGRDTLQIGNFTVRRQQFAEATK**EPGNAFLHSQFDGILGMAFP**

TAGSK **VEPVFHTLIK** **QGLVEDR** **IFSFFLNRDPNGDLGGEMILGGWNDYYFDSSK** INYIR **LSK**

VDKWQFK VDSISSTNK**TMFCK** **DSCEAIADTGTTMIIGPTDEIAAIHK** **YIGAVQNYGMGTVK** C

KDVPNLPPITFLINGTEYVLK**GK** **DYILNFDYDPYNCITGFSGVELK** **GKPWILGDVFLR** K **FYT**

IFNVQDK **AVAFATLNK** LL*

Amino acid sequence of R4FKP9. Peptides sequenced by MS/MS are highlighted in bold and underlined. Peptides encoded jointly by two exons are indicated.

- **Sequence alignment**

```
R4FKP9 MKLIFVFLVFSPIFYGSAAHVGKVQLYRRQPKSYEQYLNENIAYRDALSQLINLKSSGHS
T1I3T5 MKLIFVFLVFSPIFYGSAAHVGKVQLYRRQPKSYEQYLNENIAYRDALSQLINLKSSGHG
T1HJV8 ------------------------------------------------------------

R4FKP9 SDTVDLRNFANSQYYGSISIGTPPQSFEILFDTGSTLLWVPSFDCFSPACMTHRKYDHIK
T1I3T5 SDTVDLRNFVNSQYYGSISIGTPPQSFEILFDTGSTLLWVPSFECFSPACMTHRKYDHIK
T1HJV8 ------------------------------------------------------------


R4FKP9 SSTYTPENLEISIIYGKGSMTGILGRDTLQIGNFTVRRQQFAEATKEPGNAFLHSQFDGI
T1I3T5 SSTYT-------------------------------------------------------
|T1HJV8------------------------------IGNFTVRRQQFAEATKEPGNAFLHSQFDGI


R4FKP9 LGMAFPTAGSKVEPVFHTLIKQGLVEDRIFSFFLNRDPNGDLGGEMILGGWNDYYFDSSK
T1I3T5 ------------------------------------------------------------
T1HJV8 LGMAFPTAGSKVEPVFHTLIKQGLVEDRIFSFFLNRDPNGDLGGEMILGGWNDYYFDSSK


R4FKP9 INYIRLSKVDKWQFKVDSISSTNKTMFCKDSCEAIADTGTTMIIGPTDEIAAIHKYIGAV
T1I3T5 ------------------------------------------------------------
T1HJV8 INYIRLSKVDKWQFKVDSISSTNKTMFCKDSCEAIADTGTTMIIGPTDEIAAIHKYIGAV


R4FKP9 QNYGMGTVKCKDVPNLPPITFLINGTEYVLKGKDYILNFDYDPYNCITGFSGVELKGKPW
T1I3T5 ------------------------------------------------------------
T1HJV8 QNYGMGTVKCKDVPNLPPITFLINGTEYVLKGKDYILNFDYDPYNCITGFSGVELKGKPW

R4FKP9 ILGDVFLRKFYTIFNVQDKAVAFATLNKLL------------------------------
T1I3T5 ------------------------------------------------------------
T1HJV8 ILGDVFLRKFYTIFNVQDKAVAFATLNKSASRPITNFTPLYPGNNGASTLTNQMTHWIIL


R4FKP9 -----------    390
T1I3T -----------    125
T1HJV IICLRKWFLLV    281
```

3) R4FJC3

- **mRNA sequence**

RPRC006759

Unannotated exon

```
ATGGCTTGGAGATTAATGATTTTCCTGGTGTACTGTTTTACTCATAGTATGGCCAGCGACTCTTTGGAAAACGAT
TCAAAAAACATCTTAGAGATTCCTTTGATAAGAATGGAAAGTGCTTTACAACGAATGGCTCGAGAAAACATAAAT
CCAAGAGTTATTGAAGCTTTTTTTGAGAAAGCTGCCAAAAAGGAAAATAGTTTAGCACCAGTACCCTTATTTAAA
TTCCTGGATACTGAATTTTATGGAGAAGTGTTAATTGGTCATCCCGGCCAAAAATTTAAGGTAGTGTTTGATACT
GCATGGGCCAATAGTTGGGTACCATCTGTGTTATGCCCTGTTATACAAATTGCTTGTGCTTTACGTTCAAAATAT
GATGCTTCACGTTCGTCAACTTATGTGAAAAATAATACACCTTTTGAAGTAAAGCTTGGTTCACTAGAGCTAAAA
GGAAAATTATCAACTGATTTATTTCAGGTAGGGCAAGTAAATGTTACAAATCAAACATTTGCAGAAATAAATGAA
ATGCCATGGATTTTATTTTTTAGTAAAGTAGATGGCGTAGTAGGCCTGTCTTTCGCTGATTTTGCTATCGATGAG
GTGGTTCCACTATTTTACAATATGATCAAACAAAACGTAGTAGATCAAAAGATATTTTCATTTTATCTTAACCGT
TCTCCAACAACATCTAAAGGCGGTACTTTAATGTTTGGAGGAGTTGAAAAAAGGCACTATTTAGGGAATTTTACC
GATTTAAAAATTATTCCAAAAACAGGAGTTTGGTCTTTTCAAATTGATTGGATCTTTACAAGCAATAAAAGAGGC
CAAATGTTTTGTCAATCAGGATGTATGGCATATGCAGATACAAGTGAAAATACTATAAGAGGCCCAGCGAACGAT
ATTGAATTGCTTAATCAAATTATTGGTGCCAAATCTTTTTACTTCGGAAGATACTTTGTTCCTTGTGGTAATGTA
GCCCAGTTACCTAGAGTTACTTTCGTATTAAAGGGGAAAAAATTCACACTGAAAGGAGATGATTATGTGCAAAAG
ATGACATGGGGTCCAGTAACAATATGTTTATCTTCATTTAAAAAATCTGAAAATGCATCCGATGACAAATGGGCT
TTAGGTGTTGCATTTTTGTCAAGATATTATGTAAAATTTGATCTGGGGAAAATGCTTATTGGATTTGCTGACGCT
AGGCTTTAA
```

mRNA sequence of R44FJC3. Exons are color-coded for distinction, with odd-numbered exons in blue and even-numbered exons in gray. The genes encoding each fragment are indicated.

- **Protein sequence**

MAWRLMIFLVYCFTHSMASDSLENDSKNILEIPLIRMESALQRMARENINPRVIEAFFEKAA

KKENSLAPVPLFK**FLDTEFYGEVLIGHPGQK**FK**VVFDTAWANSWVPSVLCPVIQIACALR**SK

YDASR**SSTYVK****NNTPFEVK****LGSLELK**GKLSTDLFQVGQVNVTNQTFAEINEMPWILFFSKVD

GVVGLSFADFAIDEVVPLFYNMIKQNVVDQK**IFSFYLNR**SPTTSK**GGTLMFGGVEKR**HYLGN

FTDLKIIPK**TGVWSFQIDWIFTSNK**R**GQMFCQSGCMAYADTSENTIR****GPANDIELLNQIIGA**

KSFYFGR**YFVPCGNVAQLPR**VTFVLKGK**K****FTLKGDDYVQK****MTWGPVTICLSSFKK**SENASDD

KWALGVAFLSR**YYVKFDLGK****MLIGFADARL**

Amino acid sequence of R44FJC3. Peptides sequenced by MS/MS are highlighted in bold and underlined. Peptides encoded jointly by two exons are indicated.

- **Sequence alignment**

```
T1HRT9 MAWRLMIFLVYCFTHSMASDSLENDSKNILEIPLIRMESALQRMARENINPRVIEAFFEK
R4FJC3 MAWRLMIFLVYCFTHSMASDSLENDSKNILEIPLIRMESALQRMARENINPRVIEAFFEK

T1HRT9 AAKKENSLAPVPLFKFLDTEFYGEVLIGHPGQKFKVVFDTAWANSWVPSVLCPVIQIACA
R4FJC3 AAKKENSLAPVPLFKFLDTEFYGEVLIGHPGQKFKVVFDTAWANSWVPSVLCPVIQIACA

T1HRT9 LRSKYDASRSSTYVKNNTPFEVKLGSLELKGKLSTDLFHVGQVNVTNQTFAEINEMPWIL
R4FJC3 LRSKYDASRSSTYVKNNTPFEVKLGSLELKGKLSTDLFQVGQVNVTNQTFAEINEMPWIL

T1HRT9 FFSKVDGVVGLSFADFAIDEVVPLFYNMIKQNVVDQKIFSFYLNRSPTTSKGGTLMFGGV
R4FJC3 FFSKVDGVVGLSFADFAIDEVVPLFYNMIKQNVVDQKIFSFYLNRSPTTSKGGTLMFGGV

T1HRT9 EKRHYLGNFTDLKIIPKTGVWSFQIDWIFTSNKRGQMFCQSGCMAYADTSENTIRGPAND
R4FJC3 EKRHYLGNFTDLKIIPKTGVWSFQIDWIFTSNKRGQMFCQSGCMAYADTSENTIRGPAND

T1HRT9 IELLNQIIGAKSFYFGRYFVPCGNVAQLPRVTFVLKGKKFTLKGDDYVQKVQIIGFKL--
R4FJC3 IELLNQIIGAKSFYFGRYFVPCGNVAQLPRVTFVLKGKKFTLKGDDYVQKMTWGPVTICL

T1HRT9 ------------------------------------------    358
R4FJC3 SSFKKSENASDDKWALGVAFLSRYYVKFDLGKMLIGFADARL    402
```

4) R4FNG1

- **mRNA sequence**

RPRC014747

RPRC008989

RPRC012504

```
ATGAAAATTGCATTATTATTACTGTTCTTTGTAGGAGCTATTGTCACCAGCAATGGACAATTTAGTGTACCATTG
CGAAGAGTGCAAAAAGGGCCGAGAGATTTCAATGAATTCATGCAGACATTAACCGTTAGAAAGACCAGCCTTTAT
CATCGCTTGCTGTTGAAGAAATCCGGTGCACGATTAGACTTAAATAATAATTTAAATGTTGATTATTACGGTGAA
ATATCATTAGGAACACCACCACAAAATTTTTCTGTTATATTTGACACTGGATCTTCAGTATTATGGGTTCCATCT
TTTAGTTGTGTCCACAGCCCAGCTTGCTGGAATCACAAATTTTACTTTAGTGCAGTATCTTCAACATATCGAAGG
GACGCTAGACATTTTAAAATCGTTTATGGAACAGGAGAAGTTGAAGGACACTATTCGACTGACACGCTAACAGTA
GGTTCTCTTCAAGTGGTCAACCAAACATTTGGAGAAACAACTAAAGAAAGCGATAACCCATTTAAACACGCAAAA
TGTGATGGTTTATTTGGACTAGCTTACCCTACAGTTAGGAATGGCATCGCACCACCTCTTTATAATATGATGCAA
CAGAAACTAATTGATAGACCAATTATGTCTTTCTATCTTAACAGAAATACAAGTGCTTCCTTGGGTGGTGAATTA
ATATTCGGCGGAATGAATGAAAGTCTCGTCAAGAAAGAAACATTGAATCCCATTCCTTTAAGTAAAAAAGCACAT
TGGCAGTTTCGTATGGATGGAATTAACACGCTAACAGGAGATAATTGGTGCAAAAATGGTTGTGAAGCCATCGCT
GATACTGGCACATCCCTGATTATTGGCCCAAAATCTGATGTTTTTGGTATATTGAACAAAATTGGTGCCAAAATT
AAGAAAGGCCAAAGTGTTGGAATGGTGGATTGCAGTCGGATTTCGAAACTGCCTCCAATTATTTTCAAAATAAAC
GCCCGCAGTTACACATTGGAACCAAGTGATTATATAGTAACGATGAAATCGGGTGCTGAAAAAGTGTGCTTTGTT
GGTTTTTCTGCTTCAAGTATAAAAGATGCCCATTGGATCTTAGGAGATGTATTTTTAGGCAAATTCTACACAGTA
TTTAATTTTGGCAATGGCACTGTCTCTTTTGGACAACTCAAATAA
```

- **Protein sequence**

T1IEM8

T1HY69

T1I882

```
MKFALLHLSYYLSRKKKNLDSVPLRRVQKGPRDFNEFMQTLTVRKTSLYHRLLLKKSGARLD
LNNNLNVDYYGEISLGTPPQNFSVIFDTGSSVLWVPSFSCVHSPACWNHKFYFSAVSSTYRR
DARHFKIVYGTGEVEGHYSTDTLTVGSLQVVNQTFGETTKESDNPFKHAKCDGLFGLAYPTV
RNGIAPPLYNMMQQKLIDRPIMSFYLNRNTSASLGGELIFGGMNESLVKKETLNPIPLSKKA
HWQFRMDGINTLTGDNWCKNGCEAIADTGTSLIIGPKSDVFGILNKIGAKIKKGQSVGMVDC
SRISKLPPIIFKINARSYTLEPSDYIVTMKSGAEKVCFVGFSASSIKDAHWILGDVFLGKFY
TVFNFGNGTVSFGQLK*
```

- **Sequence alignment**

```
R4FNG1 MKIALLLLFFVGAIVTSNGQFSVPLRRVQKGPRDFNEFMQTLTVRKTSLYHRLLLKKSGA
T1IEM8 ---------------------SVPLRRVQKGPRDFNEFMQTLTVRKTSLYHRLLLKKSGA
T1HY69 ------------------------------------------------------------
T1I882 ------------------------------------------------------------

R4FNG1 RLDLNNNLNVDYYGEISLGTPPQNFSVIFDTGSSVLWVPSFSCVHSPACWNHKFYFSAVS
T1IEM8 RLDLNNNLNVDYYGEISLGTPPQNFSVIFDTGSSVLWVPSFSCVHSPACWNHKFYFSAVS
T1HY69 ------------------------------------------------------------
T1I882 ------------------------------------------------------------

R4FNG1 STYRRDARHFKIVYGTGEVEGHYSTDTLTVGSLQVVNQTFGETTKESDNPFKHAKCDGLF
T1IEM8 STYRRDARHFKIVYGTGEVEGHYSTDTLTVGSLQVVNQTFGETTKESDNPFKHAKCDGLF
T1HY69 -----------------------------VGSLQVVNQTFGETTKESDNPFKHAKCDGLF
T1I882 ------------------------------------------------------------

R4FNG1 GLAYPTVRNGIAPPLYNMMQQKLIDRPIMSFYLNRNTSASLGGELIFGGMNESLVKKETL
T1IEM8 GLAYPTVRNGIAPPLYNMMQQKLIDRPIMSFYLN--------------------------
T1HY69 GLAYPTVRNGIAPPLYNMMQQKLIDRPIMSFYLNR-------------------------
T1I882 --------------MKSKFLEKNRNFAIPYVWATRNTSASLGGELIFGGMNESLVKKETL

R4FNG1 NPIPLSKKAHWQFRMDGINTLTGDNWCKNGCEAIADTGTSLIIGPKSDVFGILNKIGAKI
T1IEM8 ------------------------------------------------------------
T1HY69 ------------------------------------------------------------
T1I882 NPIPLSKKAHWQFRMDGISTLTGDNWCKNGCEAIADTGTSLIIGPKSDVFGILNKIGAKI

R4FNG1 KKGQSVGMVDCSRISKLPPIIFKINARSYTLEPSDYIVT---------MKSGAEKVCFVG
T1IEM8 ------------------------------------------------------------
T1HY69 ------------------------------------------------------------
T1I882 KKGQSVGMVDCSRISKLPPIIFKINARSYTLEPSDYIVTVSIMRILYKMKSGAEKVCFVG

R4FNG1 FSASSIKDAHWILGDVFLGKFYTVFNFGNGTVSFGQLK
T1IEM8 --------------------------------------
T1HY69 --------------------------------------
T1I882 FSASSIKDAHWILGDVFLGKFYTVFNFGNGTVSFGQLK
```

5) R4G3V2

- **mRNA sequence**

RPRC002478

Unannotated exon

RPRC002479

Missing exon

ATGTCACAATATTGTACAGTCTTACTTTGGTTATTGTTAGCATTAACATTTAATGAACTCAATGGATC
CACCATTAAAATCCCTTTGTACCATCGTAAGAAACAGGCAAAGAGTATCGCAGACTATTTTGGTAAGT
TGGTTGATCCCATACCACTTCCGCCATTCTATACACTAAAAACTGAAAGACACCTTGAGCCACTCAAG
AACTATCAAAATGTACAATATTACGGCGTAATTAAATTAGGAACTCCTCCACAAGAGTTTGAAGTAGT
TATCGATACTGGTTCATCGAATCTTTGGGTACCATCGTCGAAATGTGTGGATGAAAGTGAAGTTTGCC
GTACTCATAAAAAATATAACAGCTCTTTGTCTACAACTTTTGTGGAAGATGGAAGAAAAGTGAAAATT
GCTTACGGTTCAGGATCAGTTAAAGGATTTTTCTCAACCGACATTTTAACACTTGCCGACGTACAAAT
AAATAATCAAACATTCACTGAGGCAATTCAAGTTCAAGGATTTGATGTTGCCAAATTTGATGGACTTC
TAGGATTGGCTTTTCCAAGTATAGCTAAGAAGCACGCGAGACCACCATTTTTCAATATGATTAAACAG
GGTTTACTGGAGAAACCATTATTTTCAATCTATCTGACAAAAATTGAGAATGATCCAGAAAATGGCGG
TGTTTTAGAATTTGGAGGCTGGGACTCGGAGAAATTCGATCCTAACGATGTGCATTATATTCCTTTAA
GCGAGCCAAATTATTGGCAGTTCTCAGTCGATACAATAAAAGTCGGCGAAACCAATTTATGCAAAGAT
CAATGTGAAGCGGCTGCTGACACCGGAACGTCACTCATTATTGGACCACATGACGAAATTGTTGCATT
GCACGAAGCTATTGGTGCTGAACTTTATAAGGATAGAATTGGCGTGATTGAATGTTCAAAAGTTCCCG
AACTACCACCAATAGTATTTACAATTCATGGCAAAGATTATCAGCTGGTACCATCGGATTACATTCAA
ACTTTCCCAGAGGAACCGAGCTATTGCTTAACTGGATTTTCCACCATGAAGGACTACAAAGGAATGTG
GCTCCTTGGAGACTTATTTCTCGTTAAATATTACACAATATTCAATGCAAAGGAACGCACCATTGCAT
TCGCTAATTTAAAAGAATAA

- **Protein sequence**

T1HEK6

T1HEK7

Missing

MSQYCTVLLWLLLALTFNELNGSTIKIPLYHRKKQAKSIADYFGK**LVDPIPLPPFYTLK**TER
HLEPLKNYQNVQYYGVIK**LGTPPQEFEVVIDTGSSNLWVPSSK****CVDESEVCR**THKKYNSSLS
TTFVEDGRKVKIAYGSGSVKGFFSTDILTLADVQINNQTFTEAIQVQGFDVAKFDGLLGLAF
PSIAKKHARPPFFNMIKQGLLEKPLFSIYLTKIENDPENGGVLEFGGWDSEK**FDPNDVHYIP**
LSEPNYWQFSVDTIKVGETNLCKDQCEAAADTGTSLIIGPHDEIVALHEAIGAELYKDRIGV
IECSK**VPELPPIVFTIHGK**DYQLVPSDYIQTFPEEPSYCLTGFSTMKDYKGMWLLGDLFLVK
YYTIFNAKERTIAFANLKE

- **Sequence alignment**

```
T1HEK7 ------------------------------------------------------------
R4G3V2 MSQYCTVLLWLLLALTFNELNGSTIKIPLYHRKKQAKSIADYFGKLVDPIPLPPFYTLKT
T1HEK6 MSQYCTVLLWLLLALTFNELNGSTIKIPLYHRKKQAKSIADYFGKLVDPIPLPPFYTLKT

T1HEK7 ------------------------------------------------------------
R4G3V2 ERHLEPLKNYQNVQYYGVIKLGTPPQEFEVVIDTGSSNLWVPSSKCVDESEVCRTHKKYN
T1HEK6 ERHLEPLKNYQNV-----------------------------------------------

T1HEK7 ---------------------------------LADVQINNQTFTEAIQVQGFDVAKFDG
R4G3V2 SSLSTTFVEDGRKVKIAYGSGSVKGFFSTDILTLADVQINNQTFTEAIQVQGFDVAKFDG
T1HEK6 ------------------------------------------------------------

T1HEK7 LLGLAFPSIAKKHARPPFFNMIKQGLLEKPLFSIYLTKIENDPENGGVLEFGGWDSEKFD
R4G3V2 LLGLAFPSIAKKHARPPFFNMIKQGLLEKPLFSIYLTKIENDPENGGVLEFGGWDSEKFD
T1HEK ------------------------------------------------------------

T1HEK7 PNDVHYIPLSEPNYWQFSVDTIKVGETNLCKDQCEAAADTGTSLIIGPHDEIVALHEAIG
R4G3V2 PNDVHYIPLSEPNYWQFSVDTIKVGETNLCKDQCEAAADTGTSLIIGPHDEIVALHEAIG
T1HEK6 ------------------------------------------------------------

T1HEK7 AELYKDRIGVIECSKVPELPPIVFTIHGKDYQLVPSDYIQTVSLEFCSNIV-FSALL---
R4G3V2 AELYKDRIGVIECSKVPELPPIVFTIHGKDYQLVPSDYIQTFPEEPSYCLTGFSTMKDYK
T1HEK6 ------------------------------------------------------------

T1HEK7 -------------------------------    203
R4G3V2 GMWLLGDLFLVKYYTIFNAKERTIAFANLKE    391
T1HEK6 -------------------------------    73
```

6) R4G4V0

- **mRNA sequence**

RPRC012508

RPRC012513 (3479-3682 PB)

Missing 4335-4580 PB

Missing 4212-4335 PB

Missing 1315-2998 PB

```
ATGAAATTTACTTTATTATTAATTTTATTCATTGGAACTGCTGTCAGCATCAGTGGGATTCTCAGGGTTCCACTG
CACAAAATGTACAAAGAGCCTAGAGAATATCATGATTCACGAAGACGGCAAAATCGCGCACAACAGTCTTCAACA
TTATTTAGAGTTGCAAGAAATGGTGTAAAGAATATTAAAAACAATTTAAATGCCGCATATTATGGTGAAATATCA
TTAGGCACTCCGCCACAATATTTTTCTGTAATATTTGACACTGGATCATCAAATTTATGGGTTCCATCTTCTAGT
TGTTACTTAAGCTTAGCCTGCTGGAATCATCAGTACTATAAAAGAAGTCAATCATCAACATATCGTAAAGATGGG
AGACATATAAAGATATCTTATGGTACTGGAGAAGTTAAAGGATTTTTATCTACGGATACACTAACTGTAAATTCT
ATTCATGTGCTTAATCAGACATTTGGAGAAATAACTTCAGAAAGTAGAGAACCATTCTGGAAGGCTAAGTTCGAT
GGTATATTTGGACTTGCGTTTCCTTCAATTGCAGCAAATGGTGTTCTACCACCTGTTTACAATATGGTATCCCAG
AAACTCATTGACACATCAATCTTTTCTTTCCTCGACAGAAACATAGGTGCTTCTTTGGGTGGTGAATTAATATTC
GGAGGTATTAATGAAGATCTAGTAATAAAAAACTCCTTAAATCCGATCCCTTTAAATAGATTAGGTTACTGGCAG
TTCATAATGGATCGAGTTAAAACTTCAACAGGAGATCAATGGTGCAAATATGGTTGTAATGCAATCGCTGATACT
GGAACTTCAATGATAATTGGACCAGAAATAGATATTTTAAATATATTCAAAACACTTAACGCCACAATCAGCAAT
GGCTTTGGAGCTGTGGATTGTGCGCAAGTTTCATATCTACCTCCAATCACTTTCGATATAAATGGACGCAGTTAT
ACATTGGAATCGAATGATTACATTTTAAGATTGAAAGACGGAGTAAAAACATTGTGTCTCGTAGGGTTCTCTCCC
GCAACGTATTCGCAAAAAATTTCTTGGATCTTAGGAGATGTATTTTTAGGTAAATTCTATACAGTATTTGATTTT
GAAAAACACACTATCTCTTTTGGTCAACTGAAATAA
```

- **Sequence alignment**

```
R4G4V0 MKFTLLLILFIGTAVSISGILRVPLHKMYKEPREYHDSRRRQNRAQQSSTLFRVARNGVK
T1I891 ------------------------------------------------------------
T1I886 MKFTLLLILFIGTAVSISGILRVPLHKMYKEPREYHDSRRRQNRAQQSSTLFRVARNGVK

R4G4V0 NIKNNLNAAYYGEISLGTPPQYFSVIFDTGSSNLWVPSSSCYLSLACWNHQYYKRSQSST
T1I891 ------------------------------------------------------------
T1I886 NIKNNLNAAYYGEISLGTPPQYFSVIFDTGSSNLWVPSSSCYLSLACWNHQYYKRSQSST

R4G4V0 YRKDGRHIKISYGTGEVKGFLSTDTLTVNSIHVLNQTFGEITSESREPFWKAKFDGIFGL
T1I891 ---------------------------VNSSHVLNQTFGEITSESREPFWKAKFDGIFGL
T1I886 YRKDGRHIKISYGTGEVKGFLSTDTLT---------------------------------

R4G4V0 AFPSIAANGVLPPVYNMVSQKLIDTSIFSFLDRNIGASLGGELIFGGINEDLVIKNSLNP
T1I891 AFPSIAANGVLPPVYNMVSQKLIDTSIFSFFLDR--------------------------
T1I886 ------------------------------------------------------------

R4G4V0 IPLNRLGYWQFIMDRVKTSTGDQWCKYGCNAIADTGTSMIIGPEIDILNIFKTLNATISN
T1I891 ------------------------------------------------------------
T1I886 ------------------------------------------------------------

R4G4V0 GFGAVDCAQVSYLPPITFDINGRSYTLESNDYILRLKDGVKTLCLVGFSPATYSQKISWI
T1I891 ------------------------------------------------------------
T1I886 ------------------------------------------------------------

R4G4V0 LGDVFLGKFYTVFDFEKHTISFGQLK     386
T1I891 --------------------------     67
T1I886 --------------------------     147
```

7) R4G2R0

- **mRNA sequence**

Missing
39710 – 39782 bp

Missing
38589 – 38756 bp

```
ATGTTCACTGGAGCCTTTCGTTTAGTGTTCCTGCTGTGTTTACTGGGAATTGTGGTCCATGCTCAGTTCAGGTTG
ACTCTTCATCGGCGTAAAATAGGCTTAAAATCTTATAGCGAGTACAGTACTGCATTAAACCAATGGAGGGAACAA
TTCCATAGGTACAAAACTTTAAAGGCTACCTCGAAAATATTAAAAGCAGCCGATTACGGAAAAGTTAAACTATTG
AATCATATGAATGCAGAATATTATGGGGAAATATTTCTAGGCAGTCCACCCCAGAGGTTCCTGGTTGTGATTGAC
ACGGGCTCCTCTGATTTATGGGTCCCCTCGAAGCAATGTTCCTTTTTTAATTTAGCTTGCTGGATTCATAATAAA
TATGATCACACCAAATCATCTACTTACCATGATGTTGGAAAAGAAATGAGAATTTCTTATGTTACTGGAAGTATG
GTGGGACGAATTGGCGAGGAAGAAGTGCGAATTGGTGGAATGCTTCTTAAAAATCAAACATTTGCCGAAGCAATA
GAAGAACCTGGGATGACATTTGTCTTTTCAGAATTTGATGGCGTTATGGGGCTGGGATTTAGTGAACTAGCTGAA
TATGGGTCGCCTGTGCATTATAATATGTACCATCAAAAATTAATATCTGATTTCGTGTTTTCTATTTATTTGAAC
AGGGATGAAAAAGACAGTTTTGGTGGTGAAATTCTATTCGGTGGTACTGATGAAACAAAATACAACAAAACTACA
CTTAGATATGTGAATTTAACTGAAACAACATTCTGGCAATTTAAATTGGATGGAATGAAAGTAGGCGATCTAAAA
ATCGATTTAGACGATGTGCTAGCTATAGCAGATACGGGAGCTTCTCTAATTATTGGTGAAACGGAAGTGATAAAT
AAGTTTTATACCACCATCGGAGCTAGCGTTGATGGAGCAGATGCTTATGTGGACTGCGACAAAGTTGATCAACTT
CCGCCAGTAGAGTTCATCATTGGTAACCAAGCATTCAGGCTGGAAGGAAAAGATTATATCATCAAAGTTAAATCT
TACATTTTCTGGACTAAATGTTTAATCGGTATTGCTGGAATGGAACTGGGAGGCGAGGAATGGATCTTGGGAGAT
GTATTTCTAGGACGATATTATACCATATTTAATGTAACTGAAGGAAGAATTGGCTTTGCAGATCGTTTGCGTTGA
```

- **Sequence alignment**

```
R4G2R0  MFTGAFRLVFLLCLLGIVVHAQFRLTLHRRKIGLKSYSEYSTALNQWREQFHRYKTLKAT
T1HJE8  ------------------------------------------------------------

R4G2R0  SKILKAADYGKVKLLNHMNAEYYGEIFLGSPPQRFLVVIDTGSSDLWVPSKQCSFFNLAC
T1HJE8  -------------------AEYYGEIFLGSPPQRFLVVIDTGSSDLWVPSKQCSFFNLAC

R4G2R0  WIHNKYDHTKSSTYHDVGKEMRISYVTGSMVGRIGEEEVRIGGMLLKNQTFAEAIEEPGM
T1HJE8  WIHNKYDHTKSSTYHDVGKEMRISYVTGSMVGRIGEEEVRIGGMLLKNQTFAEAIEEPGM

R4G2R0  TFVFSEFDGVMGLGFSELAEYGSPVHYNMYHQKLISDFVFSIYLNRDEKDSFGGEILFGG
T1HJE8TFVFSEFDGVMGLGFSELAEYGSPVHYNMYHQKLISDFVFSIYLNRDEKDSFGGEILFGG

R4G2R0  TDETKYNKTTLRYVNLTETTFWQFKLDGMKVGDLKIDLDDVLAIADTGASLIIGETEVIN
T1HJE8  TDETKYNKTTLRYVNLTETTFWQFKLDGMKVGDLKIDLDDVLAIADTGASLIIGETEVIN

R4G2R0  KFYTTIGASVDGADAYVDCDKVDQLPPVEFIIGNQAFRLEGKDYIIKVKSYIFWTKCLIG
T1HJE8  KFYTTIGASVDGADAYVDCDKVDQLPPVEFIIGNQAFRLEGKDYIIKVKSYIFWTKCLIG

R4G2R0  IAGMELGGEEWILGDVFLGRYYTIFNVTEGRIGFADRLR
T1HJE8  IAGMELGGEEWILGDVFLGRYYTIFNVTEGRIGFADRLR
```

8) R4G5J6 and T1IFK7 sequence alignment

```
T1IFK7 ------MKFALFLSLFFIGAAVSDVRILRIPLHKINREPRNYHEFVKTVTEAHNSLQRFL
R4G5J6 MYGFIKMKFALFLSLFFIGAAVSDVRILRIPLHKINREPRNYHEFVKTVTEAHNSLQRFL

T1IFK7 VLQKSGVQE-------AAYYGEISLGTTLQNFSVVFDTGSSNLWVPSSSCYFSLACWNHH
R4G5J6 VLQKSGVQEVLKNNLNAAYYGEISLGTPLQNFSVVFDTGSSNLWVPSSSCYFSLACWNHH

T1IFK7 YYKRSKSSTYIKDDSPLSIAYGTGEVEGFLSTDTLTINTLKVANQTFGEITSMSRKPFRE
R4G5J6 YYKRSKSSTYIKDDSPLSIAYGTGEVEGFLSTDTLTINTLKVANQTFGEITSISRKPFRE

T1IFK7 AKFDGILGLGYPSIATGHVVPPVNNMVNQKLIDRPIFSFYLNRNPKETPGGELIFGGINE
R4G5J6 AKFDGILGLGYPSIATGHVVPPVYNMVNQKLIDRPIFSFYLNRNPKETPGGELIFGGINE

T1IFK7 ERVQNDTLNNPIPISNQRYWQFRMDGVNTLTGDNWCENGCDAIADTGTSLIIGPPKDILG
R4G5J6 ERVQNDTLNNPIPISSQRYWQFRMDGVNTLTGGNWCENGCDAIADTGTSLIIGPPKDILG


T1IFK7 IFKTLKAAVDGRFAAVDCKAVPTLPDIVFKINARSYTLKPEDYILRVRNRGIEICLLGFS
R4G5J6 IFKTLKATVEGPFAAVDCKAVPTLPDIVFKINARSYTLKPEDYILRVRNRGIEICLLGFS

T1IFK7 FLSGSNLPWILGDVFLGKFYTVFDFGNNTVSFGQIKQIKLL
R4G5J6 FLSGSNLPWILGDVFLGKFYTVFDFGNNTVSFGQIKQIKLL
```
